# The self-reflexivity in transnational literature: Re-writing the migration in female migrant writers

**DOI:** 10.12688/openreseurope.15118.1

**Published:** 2023-02-14

**Authors:** Maria Luisa Di Martino

**Affiliations:** 1 Department of Liguistics and Comparative Cultural Studies, Ca' Foscari University of Venice, Venice, Italy, 30123, Italy

**Keywords:** transnational literature; female migrant writers; self-reflexivity; migration; human mobility; hybridity

## Abstract

The main aim of this paper is to discuss the socio-political meaning of the transnational literary production made by female migrant writers. Thus, it analyses their role in the framework of the ‘hybrid’ literary production of the 21st century in Europe, such as Spain and Italy. Moving away from the idea of national literatures, this paper investigates literature as a geographical and emotional inquiry point and friction between languages, ideas, practices, literary institutions, female authors, and female voices in today’s markets. Hybrid literature written by first and second-generation migrants and displaced people is part of a huger concept of transnational literature, which breaks down with the idea of national identity and transiting towards a new conceptualization of hybridity in the literary production, also based on the translation of writings to other languages. Based on the concept of ‘the location of culture’ and the conceptualization of the Bhabha’s ‘third space’, I will analyse the relation between the positioning of female migrant writers of 21st century and the role of hybridity and the reconceptualization of the ‘third space’ in their literary production. The preliminary findings show, firstly, the idea of reconceptualising it appears in light of the complexity of migrant people's realities and sex-gender differences. By adopting an intersectional lens, focused on the dialectic between gender and race/ethnicity and class, this paper analyses the tensions embedded in the re-positioning of four female migrant writers and their transnational experience (self)reflected in their writings. The present research contributes to the scientific knowledge in the field of cross-cultural literary studies, crossed with the migration study, through questioning the changing gender role and relations in transnational migrant literature. In addition, the findings show that today's reflection on ‘third space’ theory in the diasporic literature seems like an idea to be refined when migrant women are involved.

## Plain language summary

The present research is focused on the transnational literary production made by female migrant writers and the way they speak about their personal migratory experience. It shows another way of knowledge production by female migrant authors. This article analyses four female authors' novels: (1) Lea Ypi's ‘Free’ (2021); (2) Margaryta Yakovenko’s ‘Desencajada’ (2021); (3) Karina Sainz Borgo's ‘El tercer país' (2021); and (4) Sabrina Efyonayi's ‘Addio, a domani. La mia incredibile storia vera’ (2022). The analysis is on two levels: the text and the conversation with the authors. The main aim is to show new perspectives on migration and human mobility and show examples of real stories of migration, which can help migrants to manage their feelings about their migratory experience. In that sense, self-reflexivity is a tool to speak about complex processes of integration and adaptation in the destination countries and a new way to find solutions to inner conflicts through the writing.

## Introduction

The questions of identity formation and identity reconstruction are crucial in today’s debates concerning migration, human mobility, human rights, and gender equality policies in European countries and the United Kingdom. These topics are among the most important issues for the societal improvement and the social change. Especially, in a time in which the European Union (EU) is living difficult parallel phenomena, such as the demographic decrease and increasing aging population, on the one hand; and, on the other hand, the integration processes of migrants’, marked by the racism and/or xenophobia in the host societies. There is an urgent claim for a better understanding of migratory dynamics, based on the articulation of the micro-, meso-, and macro-sociological analytical lens ( Martiniello & Rea, 2014; Di Martino *et al.*, 2020). In addition, the recognition of the distinction between female and male migrants’ differences, opportunities, and needs, has an important role in the present-day debates about the way of reframing the positioning of migrant people in European societies. Both the knowledge production on migrations and human mobility and the migrants’ oral or written stories, are suitable for guiding and improving the EU’s integration policies and discourses, including the analysis from the gender perspective.

The field of politics for the gender equality, and policies for the integration of migrants, crossed with the literary production from migrant subjectivities and female migrant writers and approached by an interdisciplinary perspective, can help to shed light on subjective conceptualisation of the subject on the move. Moreover, they may help to reshape the idea of home, migration, displacement, places of resettlement, and the act of crossing borders, which are managing as real or imaginary. These concerns are important to understand the hidden vectors of change in our changing societies, linked with the human mobility process, as through the literary production and the self-reflexivity of migrants, and, in particular, of female migrant writers in their works, we can delve deeper into the psycho-sociological dynamics at the structural and systemic level. At the structural level, the structures of opportunities and constrains, and the structures of power and oppression, should be analysed. At the systemic level ( Kofman, 2019; Pisacane & Kilkey, 2021), the sources, elements, and factors, which produce vulnerabilities, should also be studied.

Linked to this, the writing that emerged as a self-reflexive action from female migrants’ voices and narration can be a creative reinterpretation of their personal history, of their migratory trajectory and experience. It represents the delivers of powerful reflection on migration and mobility. It is a mirror for our societies, which reflects the societal challenges of our time, that remains crystallised in literary. At the same time, it is also a challenge for the interdisciplinary sociological research and knowledge production from below. According to Smith and Watson (2010), there is a shift from the autobiography as genre to the autobiographical discourse under the umbrella genre of the life-writing as a more flexible form of self-reflexive writing. Thus, it should be stressed that in the transnational literature place and literary production are interconnected, and female migrant writers show us the duality of locality and globally places linked in the subjectivity transformation and self-reflection of their works from an intersectional approach, which reflects the intersection of different based-discrimination categories ( Di Martino, *forthcoming*). In addition, investigating the identity transformation in migration studies is also linked to other relevant issues, such as the question of negotiation of honesty of the writers and the management of the decency and politically correct discourses in the self-reflexive act in female migrants’ literary works ( Muñoz, 2022).

On the previous premises, the present article explores how migration, mobility, and literary production are interconnected with global and local changes; and how the personal female migrant writers’ experiences of migration and mobility influence the knowledge production. This topic belongs to a wider research project, ‘REWRITE: Rewriting Migrant Identities across Women’s Literature’ addressing the problem of the identity’s transformation of migrant people from a gender perspective; and the analysis of self-reflexivity in the literary production of female migrant authors between the 20 ^th^ and 21 ^st^ centuries. Among the outcomes of the project, there is the creation and piloting of the ‘Rewrite Social Lab’, which is a space for: a) the activation of the agency, related to the writing as tool for a better comprehension of personal migratory journey; and b) raising the awareness on the different problematics raised from the emigration and resettlement processes during the transit and in the country of destination. In fact, the social lab is focused on the creation of a methodology for the activation of the psychosocial wellbeing through the writing as a tool for reframing migrants’ experiences of migration and displacement.

In addition, it should be also stressed that the REWRITE project acts at three different levels, following specific objectives: a) at the micro-dimensional level, working with migrants through the extension of the uses of literature and writing as transformative tools for problem solving and a better mental health for migrant population; b) at the macro-dimensional level, the project conducts a comparative study on the influence of the policies of integration and the consequences of deconstructing and reconstructing migrant-women identities; and c) at the meso-dimensional level, searching for key element which connect the subjects’ expectations, aspirations, and desires of migrant women writers and the structures of opportunities and constrains, and power and oppression, by identifying the key elements interconnecting the two levels of analysis.

The project’s main aims are, on the one hand, the use of life-writing in the way that migrant people approach and embody the several processes of human mobility, such as the emigration and immigration; the transits; the management of the spaces in-between; and the dynamics and strategies for integration and adaptation to the host country contexts. On the other hand, to have an impact on the social change, by pushing for the integration of gender equality in the understanding of migration and mobility through another underrepresented sector, which corresponds with the literary production on migration and self-reflexivity of female migrant authors. The phenomena of migration, displacement, and resettlement are linked to the human mobility, therefore, the project pretends disseminating and democratizing the uses of literature, self-reflexivity in writing and life-writing, as tools addressed to the promotion of the social change for migrants, especially for groups which have been located in a vulnerable place, like migrant women and girls.

It should be stressed that, on the dimensions and axes of analysis described previously, the told and untold levels of exploration are also part of the analysis of the writers’ works, the analysis of the texts, and the codes linking the three main axes mentioned. To do that, I have explored, analysed, and compared different stories, different narratives, and expressive writing of female migrant writers’ literary production, who are active in different European countries (Spain, Italy, France, and Germany) and the United Kingdom. Nevertheless, in this article, I will analyse the relation between transnational literary works and social change, as well as social challenges raised from the interconnection among different voices and literary works of female writers in the frame of the self-reflexive writing. For that, in this article, I will focus on four main study-cases among the total of cases analysed for the project, selected on the basis of their relevance and presence at the present-day narrative: (1) Lea Ypi’s ‘Free’ (2021); (2) Margaryta Yakovenko’s ‘Desencajada’; (3) Karina Sainz Borgo’s ‘El Tercer País’ (2021); and (4) Sabrina Efyonayi’s ‘Addio, A domani’ (2022).

The literature written by women nowadays is still a “theoretical challenge” ( Alicino, 2015: 223) as well as the transnational literary production by female authors, which is a lesser explored field ( Estrada, 2014). The study of the diasporic literature, or intercultural literature, analysed from an interdisciplinary dimension, and approached from a multidimensional and interdisciplinary perspective, allows us to delve deeper into psychosocial problematics, vulnerability, and the structures of opportunities and constrains faced by migrants in the host country. Moreover, from a theoretical and methodological intersectional perspective, which takes into account the intersection of gender with other analytical categories, such as race/ethnicity, class, status, educational level, etc. we can delve deeper into the minority and diasporic groups’ creation of personal (micro-dimensional level) and collective identities (macro-dimensional) and belonging process construction. The relational ties, environment, and relationships are another issue (meso-dimension) in the study of migration dynamics.

In addition, it is also important to explore better the possible interconnection between the micro-, macro-, and meso-analytical dimensions of female migrants’ expressive and creative writings and the diasporic writing from the intersectional perspective, because “in these writings issues of belonging, identity, and creative expression emerge with renewed force, raising new questions about the location of production and consumption as well as the position from which interpretations and classifications arise” ( Ponzanesi, 2004:13). Related to this, to establish some clarification around the relations of power and oppression is relevant, in order to raise new questions about the diasporic literature, travel literature and resettlement, as Ponzanesi (2004) argues. At the same time, importance of the “location of production and as well as the position from which interpretations and classifications arise” (p. 13).

In this line, based on the analysis of female writers’ literature, the first question focuses on the existence of the ‘hybridity’ in this literary production, understanding the hybridity as socio-cultural phenomena given by a “conflictual positioning” ( Bhabha, 1994) between one's own culture and the culture of the colonizers. Therefore, the colonizer culture is an important element in the definition of the new identity formation processes ( Borghi, 2020), assuming the existence of a colonization heritage in migrant women’s lives. The second question I want to respond is linked with the possibility to reconceptualise the hybridity process as a space of change for female migrant writers. In that sense, it is interesting to see how the Mexican artist, Alonso Bravo, manages and represents the conflicts through his art. In relation to the transformation of the space during the human mobility process, he says that “the humanism, the transformation of the space and the transformation of the concepts of inhabiting” are three key concepts addressed to understand the international migrations” (2022). A more opened reading of interconnected processes may help us to shed light on the heterogeneity of migrant women’s stories and the different experiences of migration through the self-reflexivity in the literary production of the migrant women’s stories analysed.

Self-reflexive writing, thus, is a new instrument to study the ways to reshape heterogeneous migratory contexts and their dynamics and mechanisms. The borders are symbolic spaces of several simultaneous realities, living at the same time: decentralization and historicity. Borders, in-between places, or ‘third spaces’, are spaces in-between, marked by the temporality of the transit and the hope towards a better place. In particular, the ‘third space’ is the sociolinguistic and cultural theory starting with Homi Bhabha (1994) and addressed to the construction of a pedagogy of complexity. Its main aim is redefining cultural hybridity, which makes it possible to analyse the process of deconstruction and reconstruction of identities of subjects in transit and on the move, which is also useful for the analysis of travel literature. Among different genres, the ‘diasporic fiction’ is also dialogical, without temporality or supported on different historicity and territoriality.

The travel as existential dimension possesses a dialogic element, embedded in the constant spatial-temporal displacement. “Space is a fundamental, ineliminable dimension of existence” ( Prieto, 2019:1). In transnational literary production, it also assumes a symbolic and metaphorical dimension and it is linked with the sense of ‘home’, the concepts of ‘belonging’, and individual and collective identity, which lead to a confusion in literary production about the ‘spatiality’ and its multiple metaphors and interpretations in literary theory toward the end of the 20th century. Therefore, I would aim to establish the main axes of analysis in this study as the concepts of space as both a geographical space and an inner space of subjective experience of migration, mobility and displacement in the female migrant writers’ works. A socio-cultural re-construction is one of the main axes of analysis of the ‘transitional identity’ within the framework of the ‘third space’ theory, in the field of transnational literary production, especially focused on migration and travels. In fact, travel-writing and travel narrative construct the dialogical discourse for space-time oscillation between the macro-micro-sociological levels in perpetual becoming, today as in the past, as shown in Cervantes’ with a part of autobiographical process of his own life in his work: self-reflexivity is an important standpoint. Since one of the most important works of the universal literature of Miguel de Cervantes Saavedra (1547-1616), the existence of self-reflection in the travel-writing is evident, as well as the creation of ‘migratory characters’, as it is shown by Sáez (2019:181), referring to “el viaje de Ricote”. As Sáez (2019) argues, the self-reflexive work of Cervantes is clear; “he was a frustrated migrant” (p.181), who could not achieve the dream to travel to the Americas. Moreover, already in Cervantes, the interconnection between the travel and the national identity is evident. Thus, this is to say that travel literature is linked to the formation of national identity since Cervantes’ literary production, between 1575 and 1580 ( Sáez, 2019). From that we can learn that the travel literature is closely connected with the process of reconstruction not only of national identities, but also with the deconstruction and reconstruction process of the subjective identity ss another element to add to this equation on the personal/collective identity re-construction process, also linked with the idea of destiny.

### Conceptual framework

Transnational literature can turn into a powerful tool to explore the universe of migrant people and their migratory experiences, as it requires that we look at immigrants’ lives as a universe interconnected with home and host country and other socio-cultural networks, memoires, and personal and collective identities as part of the deconstruction and reconstruction processes. From an interdisciplinary and systemic perspective, the whole lives of migrants can be analysed from a more complete gaze, as they are to be understood as multidimensional bodies, moving in-between geographical and emotional spaces, not only linked with push and pull reasons for emigration and resettlement, as home countries are also part of their present social and cultural space in their new environments. According to Goldmann (1996), the cultural creation is another expression of human behaviour moved for the same rules of human behaviour, in which the structures are fundamental concepts to understand the totality of the reality, which is changeable constantly in the relation and interconnection both with human behaviour and the structures. Goldmann’s (1980) sociology of literature theory and methodology can help to understand the construction of knowledge in migration phenomenon and literary production by migrant people in relation to the socio-political structures. In that sense, our reality and communication are related through history; thus, the understanding of historical happenings and literary production, in the case of this research, can help to explore the relations between time, space and the human behaviour.

In fact, in Goldmann’s (1996) genetic structuralism the structure is the base of each literary work, therefore, it has to be contextualised historically and sociologically. In that sense, the positioning both of the literary work and the writers’ positionality in a specific group, linked with the specificity of their gender, race/ethnicity, class, etc., in correspondence with the claims of the intersectionality ( Crenshaw, 1989; Yuval-Davis, 2015) is fundamental determinants in the understanding of the totality of the reality lived by the writers and the historical time. The genetic structuralism, finally, helps in looking back to look forward at the harmonisation process to find replies to human beings’ behaviours and concerns ( Sefchovich, 1977:733). Therefore, the sociology of literature can study human behaviour as another sector itself of knowledge production and understanding process of reality ( Goldmann, 1996). The complexity and complexitisation of reality, in fact, is one of the ideas at the bottom of the genetic structuralism ( Goldmann, 1996). From this perspective, actually, the migration and mobility in transnational literature can turn into means to investigates the universe of literature of travels, and self-reflexive behaviours of languages, authors, readers, and the library production’s market. At the same time, it is conceived as a place and space of exchange, contact, or conflicts, between different ways of life, state of mind, and cultural and cross-cultural practices. In that sense, female diasporic writings (and the questions raised) “reveal the changed relation between cultural, historical, and political events and their representations, between the residual ideologies of colonialism and their impact on present society, and between the text and its context” ( Ponzanesi, 2004:13).

By contrast, hybridity is defined as a space in-between. The problem of definitions of the ‘third space’ and ‘hybridity’ is the target to be integrated. I have found a gap in the diasporic female authors’ hybrid production in the relation of the texts and the personal experience of migration/mobility; as well as the mismatch between identity and belonging and their definition of their production as hybrid literature. In fact, if, on the one hand, the theory of the third space overcomes the postcolonial thinking based on the dissociation of the duality between Orient as object and Occident as subject, since *Orientalism* (1978) of Edward Said, through the suggestion that both “the colonial power and discourse is possessed entirely by the colonizer” and that the colonial subject is constructing on the basis of a position of conflict ( Bhabha, 1994) through the socio-cultural domination of the colonized countries ( Bhabha, 1994). Nevertheless, Bhabha’s theory left unsolved and unproblematised the question about the “agency” ( Easthope, 1998:341). Therefore, the question of positioning is fundamental in the study of migratory trajectories and literature of travels, analysed from the gender perspective, being the experience of migration and mobility as different for men and women. In fact, it should be stressed that the literature written by women is produce from specifics spaces and places in time ( Estrada, 2014). In transnational literature, the categorical axes of analysis are centred on intersectional categories (gender, race/ethnicity, class, etc.) to those categories, others such as migrant; home/host countries, can be added for the analysis from a situated context. In fact, the situated intersectional perspective influences the way we see and know the world, with this theoretical and methodological approach of “seeing everything from nowhere” ( Haraway, 1988:189). It is linked with the “situated gaze, situated knowledge and situated imagination” ( Yuval-Davis & Stoetzler, 2002). In correspondence with the genetic structuralism theory and based on its claim to make the reality more complex, the intersectional perspective delves deeper into the exploration of that complexity. In that sense, according to Yuval-Davis (2015) “the intersectional analysis should be applied to all people and not just to marginalised and racialised women, with whom the rise of intersectionality theory is historically linked ( Brah & Phoenix, 2004; Crenshaw, 1989; Hill Collins, 1990). Finally, another element, which I shall not analyse in this paper, is the translation. I will not analyse it, due to the extension of the topic, which is related to the literary studies field. In fact, it has referred as the act of mediation between the authors and the readers ( Núñez, 2012), and translation is a field of study itself, with many theoretical and methodological approaches, and it would divert us from the main topic of this paper. However, it would be interesting to take in consideration for further studies and analyses. By contrast, in the migratory experience of migrant, refugee, and exiled people brings the translational act of a metaphorical, middle, or limbic land, where one was and is no more; where one is not yet and will be. Becoming is the constant brand. Thus, dynamism is a key element for understanding among culture.

Self-reflexivity in female’s migrant writers is a key concept underexplored on the wave of meta-theoretical theories of constructivism. It is important to stress that the core characteristics of constructivism is to be a “metatheory that can be characterised as: (1) being particularly sensitive to the distinction between the level of action (proper), the level of observation and the relationship between the two; (2) having an epistemological position which stresses the social construction of meaning (and hence knowledge) (3) having an ontological position which stresses the construction of social reality (and hence includes power)” ( Guzzini, 2000).

The concept of self-reflexivity in writing is based on Mruck and Breurer’s (2003) contribution that an aesthetic expression exists in such (…) self-reflexive patterns, and a metafictional awareness reflected in metafictional writing, as it is reflected in the textuality and the forms and strategies to construct the text. On the one hand, the study of self-reflexivity is an epistemological strategy in order to fix and explore the changeable selves of the authors and writers, basing this claim on Descartes’ *“cogito ergo sum”* (1637) in his ‘Discourse on Method’. On the other hand, Luhmann (1996) argues that self-reflexivity is the means of subjects to understand, reflect, and assess the world and their role in the society, as well as the functions of the systems that they belong. Moreover, the changes forces are based on that individuals’ capacity of self-reflection. This concept is important because its use in the piece of literary works helps to legitimate the authors’ worlds, universe, and their contexts. In fact, self-reflexivity in the texts is not only a narrative strategy, but it can be approached like a positioning of the author, as it brings both the *weltanschauung* ^1^ and the *lebenswelt* ^2^ or the positioning of the author and the way to understand writers’ work. In addition, in female writers and migrant writers it is also important to analyse the system of believing and the socio-political and historical context in which they are settled. Moreover, another definition of self-reflexivity is Huber’s one: “Self-reflexivity denotes the ability of the individuals of a social system to reflect on and evaluate both their conception of the system and their role in it (…)” ( Huber *et al.,* 2005).

## Methods

In the Rewrite project, the method of sociological analysis of literature is not based on the mere analysis of texts, but it integrates the analysis of the female writers’ own experiences of migration and the study of how they have integrated and self-reflected their experiences in their works, based on the study of self-reflexivity from a multidimensional approach. Moreover, the use the interdisciplinary approach through the comparison with art productions is a cross-cultural comparison way of delving deeper into the reconstruction of meanings of female migrant authors. In fact, by crossing two different axes of analysis, such as the body of the text (the authors’ production) and the personal migratory experience of the writers interviewed. Thus, life and writing are two different level of analysis, which I have crossed to delve deeper into the self-reflexive of the personal migratory experience of the writers. To that end, I used a flexible qualitative methodology and adopted an interpretative approach in order to better understand the interplay between the personal biographies of the authors and their works, which shift constantly from auto-biographical to autobio-fictional works (see Table 1). The double analysis (on the texts and their authors) is important to understand the narrative strategies employed by the authors to tell their migratory story, based on the present and past event. Linked to the revision of the past events, actually, it is important to recognize the role of memory in the storytelling deconstruction and reconstructions strategy.

**Table 1.** Selected female migrant writers and their books included in the study.

Book Title	Author	Pub.Year	Book Editor	Origin	Country of Transit	Host C.	Reason for Emigration	Author's Profile
*Free*	Lea Ypi	2021	Bompiani	Albania	Italy	UK	Political/Crisis	Academic Professor of Philosophy in UK
*Addio, A domani.* * La mia incredibile * *storia vera*	Sabrina Efionayi	2022	Giulio Einaudi	Nigeria	N/A	Italy	Poverty	Student of Political Science/Activist
*Desencajada*	Margaryta Yakovenko	2021	Caballo de Troya	Ukraine	N/A	Spain	Work of father	Journalist at 'El País'
*El Tercer País (The * *Third Country)*	Karina Sainz Borgo	2021	Lumen	Venezuela	N/A	Spain	Political/Crisis	Journalist at 'abc' Spain

*Source*: Data collected in the fieldwork ( Di Martino, 2022)

The ‘migratory career’ lens enables us to understand the sociological dimension of the female migrant writers who were interviewed and how the structure of constraints and opportunities, as well as the relation of power and oppression influence their positionality in the destination country and their way of writing, the characters, and plotline. Moreover, the ‘migratory career’ lens also enables us to understand the circumstances linked with the writers’ real life and the turning points that have a relevant role in their memories (war, conflicts, gender-based discrimination, and other kind of discrimination). This approach sheds light on the way they balance the “initial aspirations and desires with the reality faced in the host country” ( Di Martino *et al.,* 2020: 118). The analysis of their migratory trajectories has been carried out from the sociological perspective; for that, I used the analytical lens of the ‘migratory career’ as reconceptualised by Martiniello and Rea (2014) and implemented by Di Martino *et al.* (2020) in their analysis of highly educated migrant women’s trajectories on a multidimensional level. Thus, the migratory career’s method is based on a multidimensional analysis at macro-, meso-, and micro-dimensions, as follow: a) at the macro-sociological dimension, I explored the structure of opportunities and constrains and the structure of power and oppression; b) at the meso-sociological dimension, I explored the inter-subjective relations as networks, ties, couple, family, etc. and at systemic dimension in the relation with other structures in the transit between the home and the host country; and c) at the micro-analytical dimension, the personal objectives, aspirations, expectations, and desires are analysed, and the possible turning points that influence the decision making process and the self-reflexivity in the female authors’ works. Moreover, the interviews are analyzed from the retrospective angel through the life-course perspective method, combining the spatial, temporal and biographical dimensions ( Schittenhelm, 2011:103).

### Ethical statement

This research has been conducted within an appropriate ethical framework under the GDPR (2016/679), with written informant consent signed by the female migrant writers and the adoption of the ethical data management plan (DMP), under the approval by the ethics committee of the University Ca’ Foscari of Venice (approved 18 ^th^ of Jube 2021). In addition, related to this and to the data available in this article, it should be stressed that, in this research, I have used two levels of analysis: a) the public sphere; and b) the private sphere of the writers' lives. I agreed with the female migrant writers the possibility of sharing the information and data collected about the public sphere and about their works (understood as public work and person) for the publications. But I am going to refer to the information collected in relation to their private life, to sensitive data, in an anonymous format. Finally, in relation to the latter case, sensitive data will be made available with the prior permission of the four women writers whose works and lives are analysed in this text.

### Respondents’ profile and procedures

The field work was carried out between 2021 and 2022 (ongoing) ^3^. The ‘Rewrite’ project will end on April 2023. I applied the snowball sampling technique to find the authors, based on the following selection criteria: a) writers with published works (famous or not in the literary production market); high educational level (university degree, master’s, PhD); b) job-education matching; c) a minimum of three years of professional experience; and d) a minimum of four years of continuous residence in the country of destination (in order to establish the ‘continuum of stability’ criteria ( Moreno & Aierdi, 2008: 11). The sample can provide a picture of the different channels of emigration and immigration and the reasons for emigration. After that, I approached the potential candidates by email, LinkedIn, twitter, and other social networks platforms and other events, such as the 15th International Festival of Literature of Venice (in May 2022). I also approached them through different events, such as the events organised at the University of Oxford: the conversations with the writers at the Oxford Centre for Life-Writing (OCLW) (at the Wolfson College) ^4^ and the seminars about world literature at the TORCH, the Oxford Centre in the Humanities (University of Oxford) ^5^. At the same time, I also reached out to the female migrant writers through the Female Migrant Writers’ Archive at the University Ca’ Foscari of Venice (Italy) ^6^.

### Data collection

This paper's empirical data are based on seven months of fieldwork in Spain and Italy, as part of a larger project about female migrant writers’ migration. Between March 2022 and October 2022, the fieldwork yielded data from 30 in -depth qualitative interviews conducted in Italian and Spanish and I am in charge of translations when required. 30 female migrant writers were questioned in all with some interviewed twice. The ages of the participants ranged from 24 to 60 years old. The main criterion for these interviewees was being writers with at least one book (novel or poetry) published and having been lived in the country of destination for one year: women are form the different settings and mostly from the middle or upper middle classes of the society of their home country, as they are the most capable of emigration. Initial contacts were made with them through the social networks (LinkedIn, Facebook, Twitter) I then utilised snowball sampling to contact the other female migrant writers in the five countries of analysis (Italy, Spain, UK, France, and Germany). Each interviewee was asked to read and sign the Informant Consent, before starting with the interviewing process. I adopted the ‘migratory career’ ( Martiniello & Rea, 2014) to reconceptualise their migratory trajectory through the use of self-reflexive writing in their life-writing and autobio-fiction, memoires and diaries: the central concern of this paper.

More in details, after the first contact with female migrant authors, I sent by email the informed consent form and ensured that research participants understood and consented to the export of the personal data they provided. In total, I carried out 25 in-depth interviews were held with female migrant and displaced writers from five European countries: Spain, Italy, France, Germany and United Kingdom ( Di Martino, 2022). Four of these interviews have been used for this study, the other interviews are included in the ongoing REWRITE project. I approached the writers through the social networks, such as LinkedIn, Facebook, Twitter, where I presented the project’s objective and introduced myself. I gave the link to the project for further information and I ask the writer to participate in the interview’s process. When they came back to me with a positive answer, I sent the Informant Consent and the most important questions of the interview, in order that they could have time to check them and reflect about them before the interviews, as they are very busy persons. The interviews to the authors who I have analysed for this article were carried out online, mostly because the field work started during the COVID-19 era. In the cases analysed, the interviews to Margaryta Yakovenko and Sabrina Efionayi were carried out online. To begin with Margaryta Yakovenko, her interview was held on the 17 ^th^ of February 2022, just before the Russian attack to Ukraine. In addition, the interview with Sabrina Efionayi was on the 28 ^th^ of April, two days after the book out off press. Furthermore, the first approaches and interviewing process ^7^ with Lea Ypi and Karina Sainz Borgo were in their book presentations on the 26 ^th^ of May of 2022, during the ‘15° International Festival of Literature’, which is organised yearly by ‘Incroci di Civiltà’ of Ca’ Foscari University of Venice. The interviews were conducted in the Spanish language and I did not need translation, as I am bilingual.

I prepared a questionnaire in Spanish and English, which is composed by four main themes, responding to the three main topics of research: 1) the macro-dimensions and the structures of opportunities and constrains, searching for the hindering and fostering factors in their migration processes; 2) the meso-dimension, related to the inter-relational environment, inter-subjectivity; 3) the micro-dimension, related to the inner self, self-reflexivity; personal aspirations and the intra-subjective universe; and 4) the last one is about the act of writing and what it does concern, linked with the migratory experience of each female migrant writer.

After the first contact with them, I have managed the interviews in the following steps: a) I sent by email the informed consent form, and ensured that research participants understood and consented to the publication of the personal data they provided; b) After receiving the signed copies, I arranged a meeting with each individual and provided the authors with the overview of the questions and the structure of the interview ( Di Martino, 2022) previously by email, to facilitate the interviewing action, considering that they are very busy people who travel a lot, and did not have a long time to dedicate to the interview. Thus, they could read the questions and think about the answers before the interview.; and c) We managed the interviews, which were carried out both in person and by Zoom, Gmeet, Microsoft Teams, and WhatsApp video-call, depending on the situation of each author. 

After the interviewing process, I transferred the collected data securely. For that, I used a system of codification based on the country of origin and the number corresponding to the sequence of the interviews in time-order. I used anonymisation techniques to minimise the risk to data subjects in the management of sensitive personal data, and in coherence with my previous Ethics Research Report, developed in the frame of the Marie Curie Actions. It should be stressed that this research is based on the analysis of two different kinds of women profiles interviewed: well-known famous female authors and lower or middle visible migrant/refugee writers and activists. I assessed the range of risk and vulnerability in the involved subject *ex-ante* and I asked for permissions for publishing a fragment to describe them, and the permission for take a picture for publishing a piece on social networks and on the web site of the Rewrite Project during the informed consent process. We have two cases:

1) In the first case of famous authors’ works may show autobiographical data publicly, data are visible and available from their works to the society and they will not need to be anonymised, because they were part of women writers’ literary production. Nevertheless, other sensitive and risk data will be anonymised/pseudonymised later, (during the codification process, transcription, analysis, in the publications and presentations of findings and results, in dissemination and communication activities), because the interviewer can collect high-risk and sensitive data about personal women’s specific events, hard memories, harsh life conjunctures, which should be managed as anonymously, in order to preserve interviewees’ identity and privacy. The four case studies presented in this paper are in this category, with written informed consent not to be anonymised related to the public part of the migrant women as writers, public persons and example of social activists. Nevertheless, the part of the interviews related to the writers as migrant women talking about their subjective and personal migratory experience is anonymised.
2) In the second case of lower/middle level migrant/refugee women writers involved in the Rewrite Community, and subjected to video and audiotapes, any information obtained related with this study was highly confidential. The collection of data is not anonymous in early stage for the researcher, but data have been anonymised through a system of coding in a later stage of transcription, analysis, storage, publications, etc. This is part of the ongoing REWRITE project and this data is not included in this paper.

Qualitative data that was collected and managed remain confidential and they are disclosed only with their permission. Data is not collected in an anonymous way by the researcher in the early stage of the interviews. By contrast, data are anonymised/pseudonymised at a later stage. Confidentiality is maintained by means of a code number to let the researcher of the present study know who the informers are. When the study will be finished, I will ensure that the lists, files, data bases, the names, and the whole correspondent information collected, will be completely locked down and destroyed after at least ten years since the end of the present project, as I agreed in the Ethics Report. The data collection was carried out by transcriptions and audio registration of the interviews with my mobile phone. Transcriptions were handwritten and in Spanish before being translated into English. After that, I have transcribed *verbatim* the interviews and I have anonymised the personal data of the authors with ethical approval.

The analysis was carried out based on the operationalization of qualitative information and data, through the categories linked to the concept of hybrid identities and self-reflexivity, as the main concept in the sociological analysis of the authors’ and their writings. Nevertheless, from the analysis emerged different categories, such as the political action of migrant women. They are organised in macro-, meso-, and micro-sections.

Among the total sampling, I have selected the four following case-studies (see Table 1): from Spain, the Ukranian writer, Margaryta Yakovenko and the Venezuelan, Karina Sainz Borgo; from Italy, the Italo-Nigerian, Sabrina Efionayi; and from the United Kingdom, the Albanian writer, Lea Ypi, based on the difference of migratory trajectory and the different way of arriving to the correspondent destination countries. For example, Margaryta Yakovenko emigrated with her mother, and her father was already in Spain for reasons of work. Karina Sainz Borgo came with her family when she was adult, for escaping from the Venezuelan conflict situation. Sabrina Efionayi was born in Italy (Castelvolturno, Napoli) and she can be considered a second-generation migrant. Finally, Lea Ypi emigrated to Italy to study at a university in that country, but she came after her mother and brother escaping together from Albania to Italy. These authors’ stories are embedded in different regimes of mobility ( Di Martino *et al.,* 2020; Glick Schiller & Salazar, 2013), which would influence their migration patterns.

“The term ‘regime’ suggests how national administrations affect individual mobility ( Glick Schiller & Salazar, 2013). Firstly, nationality is a key variable that establishes who is entitled to what type of mobility. In addition, the possibilities granted to certain nationality-holders depend on their economic power or class position” ( Di Martino *et al.,* 2020: 118).

 In fact, they have heterogeneous way of reconstruction of their migratory careers and migratory stories. On the other hand, I have selected these case-studies based on the different narrative strategies they adopted in their works. In fact, Margaryta Yakovenko and Sabrina Efionayi adopted an autobio-fiction strategy; Karina Sainz Borgo adopted a fictional narrative strategy and Lea Ypi adopted an autobiographical approach through the historical transition of Albania till the end of the Stalinist regime.

## Results

 In the present section, I will share the preliminary results from the field work. Open-ended questions related to the three level of analysis and questions related on the relation between author and their writing were led and transcribed handwriting and *verbatim.* The data were analysed thematically, based on the interplay of; a) constraints; b) opportunities; and c) coping strategies. They were organised in macro-, meso-, and micro-dimensions (see Table 2). In the macro-analytical frame, I found that the structure of opportunity and constrains are linked to the inter-subjective and intra-subjective elements, which guide the ‘migratory careers’ of the female migrant writers and their self-reflexive works. I have used the emic and etic approach. The emic approach allows me investigating how female migrant writers think and to know their positionality in order to write their literary works; and the etic approach allows the researcher to stress other categories of the sociological analysis, which emerged both from the female authors’ interviews and from their books, which can be used for the universalisation of different socio-cultural categories, such as the political actions in their vocational writing; the gender sensitive approach for struggling against gender-based discrimination; the anti-racist approach, to fight against the gender plus race based discrimination.

**Table 2.** Impacts of the structures of opportunities and constraints at the macro-, meso-, and micro-levels.

Level	Constraints	Opportunities	Results and strategy
**Macro**	Regimes of mobility Gender discrimination Conflicts Violence	Emigration has an instrumental meaning Study abroad	Autobio-fiction to universalise common topics
**Meso**	Isolation Melancholia Nostalgia	Make Migrants Networking Help as Mentor in the Migration and Refugee contexts	Writing as a weapon to make good things To help others With therapeutically effects
**Micro**	Self-reflexivity and Reflexivity	Writing and Rewriting authors’ own lives	Auto-biographical writing, memoires, autobio- fiction and fiction

Source: Data collected by the researcher during the field work (2021–2022) ( Di Martino, 2022).

### Hybridity

The analysis carried out shows that the conceptualisation of “hybridity” ( Bhabha, 1994) in female migrant writers’ literary production is a non-functional concept. In fact, it allows us to overcome the postcolonial duality between Orientalism and Occidentalism, and the literary production is constructed on the basis of a position of conflict (shared between colonized and colonizer). Nevertheless, in the case of the works analysed of female migrant writers, it can be said that ‘hybridity’ is reduced to a superficial discourse, when we analyse the positioning and situated contexts of origin and destination countries of female migrant writers. In fact, delving deeper into their works, we can see that the subjects’ identities on the move overcome the concept of ‘hybridity’, in order to give importance to their role as agent of change. In that sense, the female migrant writers interviewed assume, directly or indirectly, a proactive role in the storytelling of their own narratives about the migration process, the migratory experience, told in first or third person, not only inside the text, but also outside the texts, as a vector of change.

“I think that with my work I can be an example for other women on the move, I have narrated my own experience of migration and I know that other migrant women can reflect their own experiences in my own words, as they write me often about it” (M1)

In fact, they assume a role of leadership in their world, as they are, often, journalists, emigrated, refugees, or exiled from their country of origin. Their leadership is based on the construction of a counter-discourse and they are aware that their writings are a psychological help for other women in their same situation.

“I know that in a psychiatric centre the doctor use my book with his patients from different origin to work with them, and the book is a mirror where they can reflect their migratory experiences and start understanding them” (M1)

Diasporic spaces allow for the representation of those who straddle two or more cultures, languages, and ethnicities and offer a way of rethinking ( Ponzanesi, 2004). As Ponzanesi argues (2004:15) it exists a “dissymmetrical relocation” not only between the culture of origin and the culture of resettlement, but also between two different cultures in the same destination country, as the case of the Mexican and Peruvian writers in France. It is important not to presume that their locations in time and space or their modes of representing the postcolonial experience are equal or interchangeable.

By contrast, in the case of the book ‘Addio, A domani’ of Sabrina Efyonaiy 2022, it can be argued the opposite to the previous argumentation on hybridity, which is inexistent in Margaryta Yakovenko’s ‘Desencajada’ or Karina Sainz Borgo’s ‘El tercer país’. The book ‘Addio, a domani!’ is a semi-biographical work, constructed on two different periods of time and two kinds of memories: the past for her mother’s and the present for Sabrina’s story, and the memories about her origins and home country, Nigeria, transmitted to her by her mother. This book is an autobiography written in the third person, and it talks about the relation between a mother and a daughter. This the story of Sabrina, but it is also her mother’s migration story. She started with the idea to write a pure autobiographical work, but while she was writing, she realized that she could lead with her own story of her life in the first person. Therefore, she decided to change the literary narratives towards a semi-fictional telling, based on her personal experience. In this book, writing is an act of revenge against the problems the protagonist had with her mother. She has been living all her life with an adoptive family, chosen by her birth mother. Her mother was working for a woman, who exploited her, when she became pregnant. When Sabrina was born, the woman asked her mother to give the baby away. So, Sabrina’s mother asked this family of neighbours to raise the baby, in that way, she ensured that she would be able to see her daughter as she grew up, even if she was unable to raise her.

In this book, ‘Addio, A domani’, constructed on the double discourse of Sabrina on her mother’s migration and Sabrina’s research for her own identity, half-Nigerian and half-Italian, it can be argued that the “hybridity” as conceptualised by Bhabha (1994), and reflected on the dilemma between colonized and colonizer, can be approached in her experience of migration as second-generation migrant. This conceptualisation of ‘hybrid’, thus, can be translated to the relationship between Sabrina and her mother. In that sense, the conflict is constructed on the second generation of migrant’s sense of guilt. Sabrina feels ‘colonized’ by her mother’s way of thinking and cultural background. In fact, Sabrina has tried all her life to fight against this feeling of guilt, for not being totally Nigerian, and not totally Italian. On this, Sabrina tells of her internal conflict, in which she struggles daily to be herself, to encompass all identities, to overcome this hybridism and to be one: just herself.

“I was searching for my own identity, I delve deeper into the sense of [author’s maternal nationality] identity, which was very important for my mother and the sense of [author’s adoptive nationality] identity, which was my own feeling transmitted by my [author’s adoptive nationality] family (…)” (N1)

By contrast, the conceptualisation of hybridity in relation to the migration story needs to be overcome by the protagonist. The story, in fact, is a way to solve the conflicts with her mother, to overcome that dilemma transmitted by her mother’s cultural background as Nigerian women, and the conflict for desiring to be an Italian woman. For that, Sabrina uses the resilience raised from the writing to overcome her duality, and the inhibitions and rejection imposed by her mother, through the construction of her own identity, not hybrid, but proper unique identity of an Afro-Italian girl empowered for speak out by herself.

### Political action in female migrants’ writers

Studying the effects of policies on people is part of the evaluation process of positionality of evaluation, policy implementation of gender equality policies, and the importance of female actors for the transformative action of public policies is a key issue, with the obstacles and resistances they have found. It is important to explore how the social actors act in the reality of structural systems of opportunities and constrains through space and time, in order to understand how the changes can act, both at the individual and collective level. Hence, in this task, I have analysed the political dimension of female migrant writers, in order to explore their behaviour related to the human movements. In that sense, writing in the case of female migrant authors is more than a mere narrative of the facts or stories, independently from the genre selected by the writers. In a complete vision, based on the multilevel analysis of the positioning of the writers through the analytical lens of the ‘migratory career’ and the adoption of the multidimensional macro-, meso-, and micro-psycho-sociological perspective, the different levels are interconnected and a systemic relationality can be seen in the development of the literary works. On the other hand, in their writings, directly or indirectly, a personal or societal challenge, a political action, are present. Thus, directly or indirectly, the writing turns into a combination of political aspects and elements crossing the personal decision-making processes, the structure of opportunities and constrains, and power and oppression. In that sense, the main aim of the writing is to denounce the multiple discriminations experiences, together with the gender-based discrimination experienced both in the home and host country.

Ultimately, we can stress that it is not possible to separate the personal and political dimensions in the case of female displaced writers. A clear example is Lea Ypi. In case of Lea Ypi, from Albania, in her book ‘Free’, she reflects on the history of Albania through the dialogic form of the memoir. She analyses the past and her memories about Albania since she was eleven, when “everything changed in the late 1990s and nothing was the same in the last communist outpost. But the promises of freedom and justice that were to follow the 'end of history' were shattered” (2022). Moreover, fragments of her personal diary compose the final part of the book. Lea Ypi reflects about her own reality as a child, innocent and unconscious about the limitations that the regime imposed her family. She had to transform her best-known reality in another rawer version and to assume and take charge of their broken illusion when Stalin died. The illusion of freedom during the Stalinist regime in Albania turns into the self-reflexive and dialogical narrative of her memories. Thus, that sense of freedom experienced sometimes was like a heavy burden. As Goldmann (1996) claims the structure is the base of the literary production. It is evident in the case of Lea Ypi’s ‘Free’, whose life and auto-biographical experience is contextualised in the specific course of her own life. It is difficult for her to separate her family life and autobiography, from the political life of the country of origin. In her narrative women’s emancipation and feminism, are strongly linked with the figure of her mother, who was a feminist activist:

“My mother detested political action and pink quotas, and felt pity for those who appealed to them. If anyone had dared to insinuate that she had achieved her position only because she was a woman, and not because she had worked for it, she would have been ready to draw the knife again. At meetings with representatives of foreign organisations, she often emphasised the one aspect of the Communist legacy of which she felt we should be proud: the rigour with which the Party had enforced gender equality without making concessions to anyone” (‘Free’, p.203)

Moreover, she also denounces the condition of women in Albania during the regime, in which the reality of the multiple burdens that women had to bear is described. It was the price they had to pay for equality, as they worked on farms or in factories, which they had to pay for with hours of travel, and they also took care of the house, carrying out tasks that were associated with the work of "servitude" (p.205). In addition, they took care of the organisation of children and their upbringing.

“They all came home tired; but they still had to prepare dinner, help the children with their homework and wash the dishes. Then they would be on their feet, preparing the next day's meal for the family. To breastfeed or make love to their husbands or both” (‘Free’, p.203).

The pact of honesty between writer and reader is strong in her narrative, except in one point: about the narrative referred to her mother, who suddenly decided to leave everything back in Albania and move to Italy with the younger son. In this point, Lea Ypi prefers not to delve deeper in family secrets, which often are covered by a veil of mystery, by the pact of honesty that maintains that stable balance between the genealogy of decency and fiction, which is decided by the author and to which the reader lets himself go by a mysterious veil. In her book, Lea Ypi describes well the break between imagination and reality in the decision-making process of migrants, which push them to move towards a place or another. She describes that the Albanian people consider Italy as the country of freedom and the place to find refuge, but this image has to face reality, which does not correspond to their imagination, desires, and aspirations. In fact, Italy for many Albanians represented that place of possibilities, with opportunities for the reconstruction of a better life.

**Figure 1.**
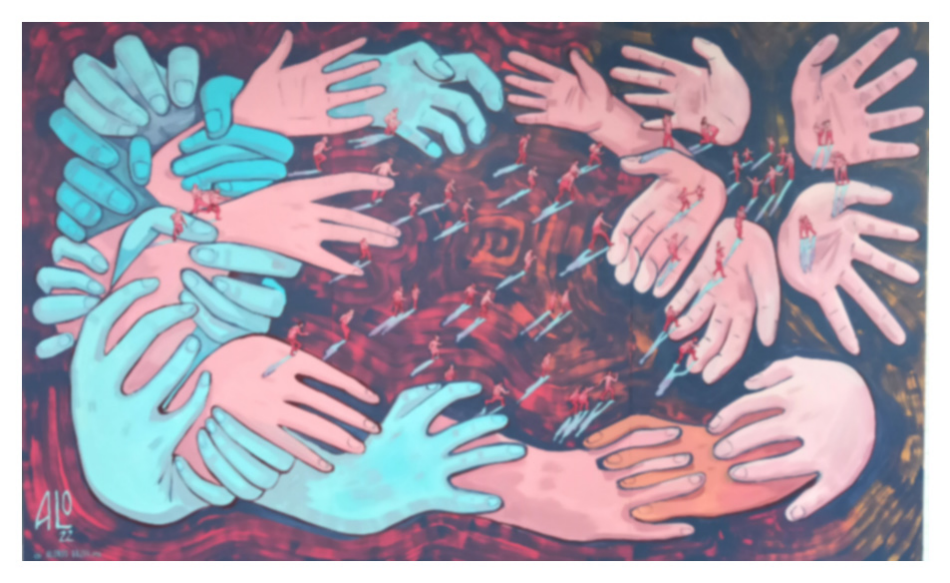
Alonso Bravo’s “The route of a Promise”. Helsinki, June 2022. This figure has been reproduced with permission from the artist.

The next book I have analysed for this study is ‘El Tercer País’ (The Third Country), of the Venezuelan writer, Karina Sainz Borgo, who is living in Spain. We see in her book a plot based on the myth of Antigone to rewrite migrants experiences of transit arriving to the borderland ‘in the middle’, called the ‘Third Country’. On the one hand, she gives their characters a universal eco, using a universal symbolism, which can be shared and applied to other conflictive realities in the world. The narrative strategies she uses are very balanced and based on the pact of a fiction with the readers. On the other hand, she uses the writing as a liberating tool, a transformative tool of her personal experience as journalist in her home country. ‘El tercer país’ is another important reference in the transnational literature. It is focused on a borderline zone, in the spaces in-between the Western and Eastern areas of a fictional country. The principal characters of this book are two women: Angustias Romero y Visitación Salazar, described as a black Virgin. The book starts with the escape of Angustias Romero and her family from the plague. She walks with her husband and her seven-month-old twins strapped to her back, who die on the way. This Figure 1 evokes an apocalyptic image of migrants who cross deserts and seas to reach a promised land, the land where they will start a new life, but end up stuck in limbic places, such as, for example, temporary refugee camps, which more often than not turn into a permanent dwelling, on the edge of humanity, of dignity. In a border space where migrants are torn between life and death. It is an apocalyptic travel-literature scenario, recalling the dangerous paths of migrants, escaping from war, conflicts and poverty; crossing the Mediterranean Sea and traveling full of hope in a new life, which for most of the time is just a dream, as they are stopped in the refugees’ camp for long time, in precarious conditions.. They are moving from the desire to have a better life, in another land, in another country, but they end up stuck in limbic places. More often, there is no return, no change; they are obliged to live on the edge of humanity, of dignity. In a border space where migrants are torn between life and death. In ‘The third country’, we can find the political dimension translated into a universal symbolism, giving a universal voice to the victims, but without re-victimize them, according with the author, she argues that her victims play an active role in the plot, in order they could reframe their lives with revenge.

### Self-reflexivity

In the case of Lea Ypi’s ‘Free’, Lea's life is centred on a double negation: on the one hand, we see her family’s life; and, on the other hand, we can read about the life of her country, in fact, the author put the readers in the middle of her era and at a personal and collective historical time. Her life and her memoire are constructed on contraries, on binary systems: socialism and liberalism; promises and broken promises; solidarity and its deconstruction; privileges and injustice. This double gaze of ‘Free’ is a half-way point between fiction and autobiographical narratives. The author uses the strategy to have an *alterego*, a substitute that makes easier the narrative of the experienced reality and memoire, embodied in different characters according to the different ages in time.

“When she becomes a teenager and prefers to dress as a boy; and the surprising Brigatista, which she gets from her father who used to watch Italian television and had sympathy for failed revolutionaries like the Red Brigades and Giangiacomo Feltrinelli. If Lea's story closes a circle, so does the fact that in Italy La Feltrinelli is the publishing house of Free” ( Michielin, 2022).

In the case of ‘The Third Country’, self-reflection is mediated by the high symbolic level of Sainz Borgo’s work, the narrative, the plot, and the characters, highly strong and impressive, is used to transform the confusing and conflicting feelings of the writer about her own country and her emigration. She said that her emigration is lived as a culpability, the same some migrant people or refugee feel as the syndrome of the Ulises as she feels herself is like a betrayal of her homeland. On the contrary, she feels integrated in Spain and well accepted in her professional and personal life.

The writers interviewed pointed out a clear standpoint about the creative process of writing about their own experience and self-reflexive processes in their works. They make, from the beginning, a clear differentiation between West and East cultures. The positioning is part of the life-writing and the expressive writing about personal and contextual facts. In this strategy, a negotiation is present with the personal identity of the author, the negotiation implies the negotiation with a personal and collective heritage, made by family’s and societal memoires and subjective or collective perceptions.

It is interesting what Tsitsi Dangaremba, from Zimbabwe, who argues that the creative process of writing in African writers is different out the creative process of writing, from Zimbabwe. She talks about the anger and the guilt she felt, and she still feels as writer who went away from her conflictive country, the same concern that I found in the conversation with Karina Sainz Borgo from Spain on her tale about Caracas, (Venezuela). Tsitsi Dangaremba asks herself “to what extent is self-reflexivity present in the novel?” ( Di Martino, *Forthcoming*) and she speaks about the different meaning between West and East. The positionality in her discourse is very important as a black women and writer, she is in the first line of the struggle for writers’ right for free speaking and writing.

Self-reflexivity is present in Karina Sainz Borgo’s narrative in the comparison with Caracas, and the migrant people’s movement towards borderline zones due to the country’s crisis. She went away from the country and she feels like a dissident and a traitor for having left her country, Venezuela, together with other colleagues, family, and friends, in a very difficult contextual time. She claims that her novels are a kind of container for letting go all the anger she had felt for that country situation and through writing she has been able to canalize her feelings; she has found the catharsis in the writing and transformation of her emotions in her literary work. In this sense, it is important to stress that, once again, the self-reflexivity of the authors in the act of writing and transforming the personal experiences. However, they have to manage and negotiate the pact of honesty on their biographical events, the writing as the reading, is another way to perform the bio-political use of the personal experience, aimed at the crystallization of that duality of fixity and dynamicity of the space and time, embedded in the constant change of a moving subjectivity in the human mobility. In the interview she said that the act of writing is a form to face her own anger and fears. In that sense, we could say that it is like exorcizing her own fears about the displacement, the hopelessness, and the death, in order to escape from the destiny of a neurosis and you can come back to reality. She affirms to liberate herself from intolerable tensions through the writing, therefore, we can argue that her writing is existentialist, in which there is a thin borderline between life and death; light and obscurity; childhood and adult world; hope and haplessness. In her work the existence of the contraries is very linked, shaping the quality of life, which recall us the surrealists and the borderline between reality and oneiric world, present in the Latin-American writers of the 20 ^th^ century.

### Structures of opportunities and constrains

Hindering and fostering factors are reflected both in the texts and oral stories of the women writers who I have interviewed. It is clear that the “situated intersectional analysis does not homogenize” ( Yuval-Davis, 2015:8), but takes into account the different positionalities of each migrant woman and writer, whose lives are intersected by different kind of overlapping categories in a constant shifting with the macro-sociological and macro-political structures of power and oppression and translated into the structures of opportunities and constraints that migrant women find in their integration process. Thus, according to Yuval-Davis (2015) “it is vital to account for the social positioning of the social agent”, in this case study for female migrant writers as vector of change, “and challenge ‘the god-trick of seeing everything from nowhere” ( Yuval-Davis, 2015:4).

According to the Goldmann’s theory and method based on the genetic structuralism, we can argue that the complexity in the deconstruction and reconstruction processes of female migrant writers’ identities and writings, as well as the self-reflexivity in their works, despite of being heterogeneous in the use of autobiographical or fictional and semi-fictional narrative strategies, is based on a common collective mental structure supported on the intersectionality as the main relational approach to the historical context reality, and the practice of the positioning both of the authors, theirs mental universes and the mental structures in a common collective subject. It means that the female authors’ lives in their migrant structured lives, expressing and living a multidimensional discrimination based on the intersection of the gender, class, ethnicity and race, status, related with the specific socio-cultural and political contexts of the country of settlement.

Structures of opportunities and constraints at a macro-sociological dimension can help to understand the behaviour of migrants and, if approached from the intersectional perspective, we can problematize more and more the reality, in order to understand better the female migrants’ situations of gradual vulnerability. In Table 2, the interconnection of the multidimensional analysis and the impact on the writers’ lives and their works is explained.

By contrast, this dialogical approach between the female writers, their works and the reality embraced, confirm the existence of that conflictual positioning between colonizers and colonized socio-cultural perception and reality creation, which is claimed by Bhabha (1994). Nevertheless, it is clear that the concept of hybridity does not explain the discrimination on the basis of gender, race/ethnicity, class, and status. The structures in the writings of migrant women writers from different countries analysed are very important in the construction of their literary production. Thus, it can be argued that stories that seem to be different belong to the same collective experience shaped by the migration and mobility. Therefore, it can be stressed that in the transnational literary production of female migrant writers are based on the collective experience of structural opportunities and constrains.

Related to this, according to Estrada (2014) and Alicino (2015) in female literature it is important to talk about the triple existing relation between women, body and writing, as the women’s body in the literary production, in general, has a transcendental meaning, which goes beyond the individual concern and go through the communitarian and political intervention ( González & Lindsay, 2022). In Alicino (2015), in fact, we can read about the existence of a necro-politics generated from the biopolitics in some Latin-American countries, such as Mexico and linked to direct and indirect violence, the exploitation of human beings, and natural resources. It recalls the Sainz Borgo’s Venezuela, linked to the movement of survival people, trapped in that ‘third space’, the borderline between the life and the death. In that sense, in the analysed work the women’s body embeds socio-political and literary concerns, confirming Alicino (2015) and Estrada’s (2014) claims.

In that sense, the writing and the literary works are linked to a common gender-based and intersectional perspective, reflected in the female migrants’ narratives, and in the representation of that sense of revenge and denounce. From these stories of women's human movements crystallised as active and living memory of migrant lives that have their own voice and express themselves and allow themselves to be heard, we can learn how they have lived and managed these trajectories of adaptation and integration. The importance of hybrid literary works and cultural creation in languages in perpetual movement, oscillating between two different cultures and languages, between presence/absence dichotomies, draw distinct mental maps that enrich the literary landscape of travel and transnational literature of new life stories, which have an educational potential, as they show us how migrant women have adaptively managed, negotiated, and transformed their identities, having a healing impact through writing as a means of mediation between two cultures. This can help to translate the experiences in practices of adaptability from a micro-psychosocial level of analysis to a meso-psychosocial analysis of the environment, contexts, and texts in which each different history is moving.

The preliminary findings also show that the transnational literature written by migrant women is based on the balance between; a) the told and untold stories; b) the negotiation between narratable and un-narratable events, following the “pact of honesty” in writing ( Muñoz, 2022); c) the development of the strategies for the plots; and d) the role of biographical and fictional narratives. In writing, a kind of research of balancing personal experiences and collective thoughts, desires, and concerns, exists. It is also a form of subversion, or a form of denunciation. In this case, in fact, the works can be considered as weapons to denounce situations and experiences of discrimination of a subject, of themselves (self-reflexive) or of a group with which they identify, or with which they sympathise based on a cause. In this type of writing, agency as denunciation is strongly revealed; in this writing, the authors set in motion mechanisms of vindication of basic human rights or the rights of entire collectives. They are not always autobiographical narratives; they can also be half-real and half fictional or auto-biofictional stories.

### Self-reflexivity in female migrant works: the three stages of the route of writing

On the base of a parallelism with the artistic mural of the Mexican artist, Alonso Bravo, entitled ‘La ruta de una promesa’ (The route of a Promise), I will explain the three stages of the route of writing in the analysed works, based on a transnational literary comparison, as part of the preliminary findings of my research. It is interesting Bravo’s artwork, as it is divided in three main parts and on the basis of this deconstruction of the migratory path, I have developed the different stages of the route of writing. The three parts of the Bravo’s work are the fight, the journey and the arrival. Those three parts represent and express the three main steps that I have found in the female migrant writers’ works analysed, as the migratory processes are in a constant developing movement both in the space, time, and at a more psychological level. Hence, this route reflects the three main stages that I have found in the texts of female migrant writers analysed, in which all the parts are intertwined with this discourse of that I call the migrant women’s ‘flight’.

First stage is the women’s flight. The first stage of women’s route in search for better life conditions, and the desire to fill their personal and professional aspirations, is the flight. This women’s flight is based on political, cultural, and structural difficulties in the country of destination, where they have few facilities to develop their own personal and/or professional aspirations, where they suffer continuous gender-based discrimination, inequality conditions’ or where they have to face the impossibility to develop their lives due to instable and insecure political and economic situation of their countries of origin; to armed conflicts, citizen insecurity, or poverty.

The second stage is the transit, which corresponds to the journey at different analytical level: a) at a subjective dimension (micro-sociological dimension); b) at a relational dimension in the host country environment; linked to the networks or ties, and also related with the inter-subjective copying strategies for integration; and c) related to the influence of the structures of opportunities and constrains and power/oppression at a relational and systemic level (macro-sociological dimension).

The third stage is the arrival. In this stage, migrant women start with a reframing process in the host country contexts, in which they move through transformative processes through the negotiation at macro-, meso-, and micro-sociological level. They also create a new space in-between. The struggle for reframing and reshaping their personal, professional, and family lives in another country, by constructing another life on the basis of the identity and cultural negotiation with the host country contexts. Moreover, the personal challenges are linked with the whole journey, the transit that embodies the transformation of the migrant women’s lives; finally, the third stage is the arrival of migrants.

### Typology

According to the genetic structuralism ( Goldmann, 1996), applied to the female migrants’ works and lives, it can be confirmed that the complexity of reality in the construction of their works is based both on the subjective mental structure, which is crossed with the historical context and society of the destination country of resettlement, on the one hand. On the other hand, it is also based on the binary or melting pot mental structure they have been able to acquire in difference grades and influenced by the transit between the home and host country. Moreover, the structure of the opportunities and constrains and the common collective mental structure they inherit from their countries of origin and their socio-cultural baggage is also important to shape their paths. In addition, the overcoming of the systemic barriers is another important complexity in the construction of different works as it is based on a common collective mental structure of a collective subject.

By contrast, self-reflexivity shows the importance of the reflection on our reality, the self-reflexivity in the writing and life-writing, indeed, is the act to know our reality and understand it; it is also a deconstruction and reconstruction process, a re-writing act in which the political dimension is a fundamental step and milestone. This is linked to two main reasons: a) among the writers interviewed there is a strong desire of revenge on an asymmetrical system of principals and double morals that they have to face, struggle, and denounce; and b) the necessity to have found a place in the world, in the new destination country in both the socio-cultural context and the linguistic dimension as the *weltanschauung* of different cultures. The self-reflexivity, finally, is also both an assessment of the perceptions and a reflection of the impact of the structure of power and oppression in the context of the female writers. Such structures, together with the structures of opportunities and constrains, based on the intersectional perspective and approach, have an impact on the transformation of the subjective identities of the writers. From this transformation, I have identified different kind of identities, moving in the framework of this complexity and intersectionality in the analysis of the lives of the female migrant writers and their works, in which the migratory experience is a picture of their own experiences and the experience of the world around them. Literary production is also an evaluation of both their own conception of the world, mixed with their personal vision of the reality and crossed with the experience of migration, mobility, and displacement, in general. In their works, their identity transformation process, the decision-making process, and the process of belonging, are the key issues which generate the turning points in their lives. Therefore, they represent options for the construction of an auto-fictional, fictional, semi-fictional, autobiographical and self-reflexive world, according to their personal evaluation.

Thus, based on the previous reflections, the preliminary findings show that it is possible to overcome the concept of hybridity and that it is possible to do a reconceptualization of the identities transformation. Apart from the socio-political axes, and the historical compromise of the female authors’ with their times, another relevant issue is the existentialist dimension in migration and mobility, both at a global level and understanding, and at the subjective dimension of the female migrant writers. By contrast, it should be stressed that this discourse is not homogeneous, in fact, not everybody has the same capacity for acting. In that sense, the findings show that the agency is a key element in the process of shaping the migrants’ lives. Therefore, the process of re-framing through the writing, which I call the ‘re-writing’ act, is based on the balanced dialogue of intertextual voices and their aesthetic reception and cathartic exercise, conducting to the liberation of oppressed emotions. Thus, I have outlined a typology of self-reflexive experiences of migration and migrant identity transformation in the transit and spaces in-between, recalling the “hybridity” ( Bhabha, 1994), which is formed by three main types of identities, fluids, crossed, and traumatized, as follows.

***1. Fluid identities***

According with Ponzanesi (2004), in the works analysed there is the idea of a fluid identity. It is formed by a migrant subject in line with the present-time fluidity. One writer, and a second-generation migrant, with two different nationalities says that her identity moves between two distinct nationalities:

“My identity has always been struggled between being [author’s maternal nationality], with a [author’s maternal nationality] mother, who came to visit me at home, and the [author’s adoptive family nationality] mother with who I’ve been living. I do not know anything about my mother’s migratory story. Therefore, the writing is for me the way I can canalise these holes in my own story, in order to know them and understand them, and, finally, to give them a meaning” (S1).

Linked to this fragment and reflection, a very interesting result can be resumed in the writer Ingy Muyaby worlds: “identity is not hybrid. The manifestation of identity can be hybrid, not identity”. Identity, in that sense, has fluidity. These identities are like a horizontal cultural line and the subjects swifts between one identity to another in a fluidity.

***2. Crossed identities***

Crossed identities, in which the authors maintain both the culture of the country of origin and the country of destination, is another kind of approach to the identity transformation and change lived by subjects and reflected in the writings of female writer’s tales, novels, or poetry. In this kind of mixed identity and writing, authors claim the language mixing as the highest level of cross-cultural approach to the writing, based on two different ways of thinking and *weltanschauung* in their own life and form of writing. In fact, according to the description of this phenomenon by another author, she spoke about a difficult transformation process. By talking about her identity’s development process, she cried during the interview because she was being aware about the hard deconstructing and reconstructing process of the whole migratory experience and identity transformation, with some hard feeling and high emotional level of information managed. It can be said that it does not end in a completely hybrid identity, but maintains that linguistic, socio-cultural barrier between two different worlds, the one of origin and the one of destination.

Nevertheless, this process of procedural and processual switching, based on the shifting between different socio-cultural construction of reality through the languages. Those two languages, understood as the mother tongue and the acquire language, or second language, of the country of residence and adoption, are not included in a hybrid conceptualization by the writers. Language is creator of new realities of mixed and cross-cultural identities, reflecting the contexts and the experiences lived in their literary production as the reflection of their time, space and mixed identities and nationalities.

***3. Traumatized and hidden identities***

These kinds of identities show the trauma and the need to reconceptualise the migratory trajectories. In this case, as the most delicate to analyse, we are faced with hard stories and migrant trajectories. There is the difficulty of dealing with personal history and also showing a great need and obligation to negotiate a pact of honesty with readers. The pact of honesty on which the understanding of writing as a ‘quasi-therapeutic’ tool is based, on the one hand; and, on the other hand, the necessary management of emotions, of breaking or maintaining the management of personal, family, and contextual decency. In this sense, the authors tend to do a great deal of personal work to maintain this balance in terms of self-reflexive work, to maintain a balance between the story of personal, autobiographical events and fiction as a strategy (sometimes) necessary for the narration of difficult or traumatic events.

Making a parallelism with the Mexican muralist Alonso Bravo’s work, I have found the three stages in the route of the female migrant writers interviewed; at the same time, in their self-reflexive works these stages are present at different level, as to say: in a told narrative and at an untold level of the text analysis. It can be extrapolated and interpreted from the analysis of the positioning of each female writer and the crossing between the country of origin and the destination country, as the crossing between the two cultures (origin and host country). It means that the positioning has a place within the writing and has a place from which the writers develop, reinterpret, and transform their migratory experience. In fact, the case of a Latin-American writer is different from the cases of European writers. Karina Sainz Borgo argues that she felt this Latin-American heritage in her migratory experience and she didn’t want to put it in her book “The third country”. This is the reason why this novel has a universal standing, and a universal claim, where all humankind, all migrants can find some of their own travel in her book, where the struggle for living is a constant topic as the same struggle for living we can find in the Mexican-US approaching migrants.

We can confirm that the literature written by women is still a “theoretical challenge” ( Alicino, 2015: 223) at the present-day global situation as well as the transnational literary production by female authors, which is a lesser explored field ( Estrada, 2014).

The three kinds of identity transformation identified in the lives and works of female migrant writers are linked to the situation experienced by the writers in connection with their specific migratory story and their position in the situated context.

## Discussion

In this article, I have analysed the migratory experience of four female migrant writers and their works from a multidimensional and intersectional perspective, crossing gender, race/ethnicity, and class, in the framework of the transnational migrant literature. In particular, I have analysed four cases of female migrant writers with the main aim to question about: a) the existence of a female migrant writing and literature; and b) the existence of the hybridity in this female literary production. The writings analysed in this article, (as well as those analysed along the whole ‘Rewrite’ project”), shows not only how writing is a living movement, which is based on a global and perpetual moving subjectivity; but also how writing, embedded in such a perpetual movement, emerges from mixed places, as transits in-between places. Writing and creativity are the main tools for reaching out across barriers and borders and making connections. Writing has a cathartic meaning in itself. It is a way to order the world, one self’s story; it is a form to solve internal conflicts; to facilitate the reframing of the migrant people’s life and fostering identity’s transformation processes. It is a way to restart and find a new order in the world in the context of the country of destination, it is also a way to transmit a message of hope; and for helping other migrants to find a sense of their migration, displacement experience.

It can be argued that the four cases analysed in this article show that a heterogeneous discourse exists depending on belonging (to home and host countries) and on the subjective transformative process. Nevertheless, a relevant common *file rouge* of all the experiences is linked to the gender dimension, the gender discomfort in habiting the world, the spaces, and the time. It is also linked with the perception of gender discrimination, which is worsened by the migratory experience of deconstruction and reconstruction of ties and the sense of loneliness, which is the pushing factor for the agency, the act of writing, the necessity to transform the migratory experience in response to injustice, no equality in the public policies and the structures of opportunities and constrains which, as shown by the female migrant authors in their self-reflexive works, shape their paths and their migratory trajectories. In that sense, despite of being voluntary or forced, the pain raising from the migration and the need to reconstruct one's own identity is a key element that pushes for the reconstruction of the whole concept of belonging.

Thus, the results show a reframing the imaginary interspace established between ‘home’ and ‘home-transit’ it can be argued that it is still linked to the concept of ‘belonging’ to different spaces simultaneously and the identities’ transformation process linked with that intersectional perspective in practice in their writings.

It can be stressed that the idea of reconceptualising the hybridity in female migrants’ work as insufficient categories for explaining the whole migratory experience and the systemic complexity of the interconnection between macro-, meso-, and micro-sociological dimensions, analysed both in the text and through in-depth interviews with the female migrant authors. On the other hand, it is not sufficient to embrace the socio-political and multicultural complexity of migrants’ realities, which appears and transcend the migratory and displaced experience of this group, not in their personal lives nor in their literary works. Indeed, by adopting the intersectional lens, the complexity of reality spreads from the dialectic between inter/intra-categorical interconnections of gender, race/ethnicity, class, educational level, status, etc.

In that sense, it also conducive to the need to rethink hybridity as conceptual category in the light of the intersectional perspective. It is not enough to describe the different and heterogeneous migratory experiences, nor the process of decision making, or the process of identity transformation lived by the women writers who pour into their self-reflexive literary production the concerns, the problems and the strategies used for adaptation and integration, lived as migrant women in a foreign country, in a country of resettlement.

It is important to recognize the limits established by the theory of the ‘third space’ ( Bhabha, 1994) and the conceptualisation of the hybridity in transnational literature, especially that literary production analysed from the intersectional perspective, as it does not include the intra-categorical and inter-categorical ( McCall, 2005) intersectional perspective, on the one hand; and, on the other, it does not include the point of view of the authors, the creators of cultures from another relevant perspective linked with the self-reflexivity in the act of writing and the inner scope of their writing, which in the most of cases is link with a strong agency. In fact, in the four cases analysed it can be stressed that the act of writing has two main objectives: the first one is more linked to the subjective author’s production and the inner feelings that move the writers to reframe o re-elaborate the personal migratory story and experience. The second one is considered in the relation with the historical time and context in which the writers use their voices, their writing, as the most powerful tool for deconstruction of the stories and reconstruction of meaning according to the re-frame of the internal negotiation of identity and culture in the matching process between the internal forces and strategies of adaptation and the external structures of opportunities and constraints.

Another evidence is that the female migrant writers share a common *file rouge* based on their migration as a type of exiling themselves from the home country, the family of origin, and the past where they found oppression and discrimination based on gender, ethnicity/race, class, etc. According to Ponzanesi, I have found the claim for an act “an active role in the construction of their nomadic existence, and refuses the passive role of victim of History, as an oppressive entity which cannot be challenged” (2004, p.170). Writing in this sense is a political act, based on the reproduction of the self-reflexive path of migration. It is political, also because it is based on the feminist principle, and slogan, that the personal is political, disseminated during the second wave of feminism between the 60s and 70s: ‘the personal is political’. This claim is still alive, much clearer, and stronger than ever in the female migrant writers, who are pushed to find new ways of expression for the elaboration of their personal history.

## Conclusion

This present article looks into the literary production of migrant women as a testimony of life stories, based on international transit and migration, we can learn how they have managed emotions, processes of identity transformation and socio-cultural negotiation for integration and adaptation. Literature is a perpetual movement and there is a symmetry with human mobility and migration phenomena, which are also perpetual movement in the effort to negotiate with the new universe of the host country context migrants’ own identities. Hence, we can argue that a symmetry between human mobility, migrations, and the self-reflexive literary production of migratory experiences of people on the move is constructed on this association of contraries ideas and meanings. Finally, such concerns should be taken into consideration by public polices, as it is shown by the findings, the present day’s politics demand a reframing and readjustment of migrant women’s identities in the transit and in the host country, as they have to face with several structures of opportunities and constrains, adopting copying strategies for fitting in the host society’s context.

A last remarkable finding about the action of re-writing consists in two main actions: a) to speak out about the experience of migration, through the auto-biographical, auto-fictional or fictional genre; and b) to reframe the migrants’ life and experience of migration, independently from the level of trauma that they possibly bring. The action of re-writing is the act of reconstruction of a female migrant’s identity without define what identity is, but through the deconstruction of their own definition as ‘migrant’, as ‘woman’, as ‘writer’, they have the opportunity to reframe their life through the act of writing; and to give names to their experience. The gender-based politics and psychosocial interventions, therefore, should be improved through gender-sensitive politics, both from the knowledge production and from the migrants’ stories, for guiding and improving the EU’s integration policies and discourses, including the analysis from the gender perspective.

## Data Availability

The transcripts of interviews for this study are restricted to protect personal data under the approval of the ethics committee of the University Ca’ Foscari of Venice (approved 18th of June 2021) under the ongoing ‘Rewriting Migrant Identities across Women’s Literature’ (REWRITE) project. Once the study has been completed in April 2023, all the underlying data will be completely locked down and destroyed at least 10 years after the end of the project. To request access to the underlying data, researchers are required to contact the corresponding author Di Martino Maria Luisa ( marialuisa.dimartino@unive.it) and the DPO of the Projects: Lawyer Giorgia Masina, University Ca' Foscari of Venice Ethical Committee ( dpo@unive.it) and provide a detailed explanation as to why they wish for access to the underlying data. Transcriptions cannot be publicly shared or published, access to the data will be provided if permissions are obtained from the female migrant writers' interviewed and the ethical committee for this study. Zenodo: Questionnaire Blank Copy English. https://doi.org/10.5281/zenodo.7264700. ( Di Martino, 2022a). This project contains the following extended data: Rewrite_Interview.docx (Blank copy of the interview questions used in this study in English) Zenodo: Title of project. https://doi.org/10.5281/zenodo.7330790. ( Di Martino, 2022b). This project contains the following extended data: Rewrite_Interview_SPANISH.docx (Blank copy of the interview questions used in this study in Spanish) Zenodo: Privacy notice templates. https://doi.org/10.5281/zenodo.7277459. ( Di Martino, 2022c). This project contains the following extended data: PRIVACY NOTICE TEMPLATES_EN. (Informed consent form in English). PRIVACY NOTICE TEMPLATES_ES (Original informed consent form in Spanish). Data are available under the terms of the Creative Commons Attribution 4.0 International license (CC-BY 4.0).

## Footnotes

^1^ Worldview (translated by the author).

^2^ Living environment (translated by the author).

^3^ The estimated date of the field work is the end of December 2022.

^4^ https://www.wolfson.ox.ac.uk/oxford-centre-life-writing-oclw

^5^ https://torch.ox.ac.uk/

^6^ https://www.unive.it/pag/27331/

^7^ After the first step, I have followed the interviews’ second step online in June with Karina Sainz Borgo, and in October with Lea Ypi (which is ongoing).
