# Supplementary material for: The self-reflexivity in transnational literature: Re-writing the migration in female migrant writers
Source: Open Res Eur. 2023 Jul 31;3:31. Originally published 2023 Feb 14. [Version 2] doi: 10.12688/openreseurope.15118.2 (PMC10450254; doi:10.12688/openreseurope.15118.2)
Supplement: Peer review history

# Peer review: Reviewer response for version 2

**DOI:** 10.21956/openreseurope.17590.r34106

**Published:** 2023-08-24

**Authors:** Lucia Miranda Leibe

**Affiliations:** 1 Universidad Católica Silva Henríquez, Santiago, Chile

I confirm that I have reviewed the comments and responses provided by the author, and I do not have any other suggestions to add.

If any, are all the source data and materials underlying the results available?

Yes

Is the work original in terms of material and argument?

Yes

Is the work clearly and cogently presented?

Partly

Is the argument persuasive and supported by evidence?

Partly

Does the research article contribute to the cultural, historical, social understanding of the field?

Yes

Does it sufficiently engage with relevant methodologies and secondary literature on the topic?

Yes

Reviewer Expertise:

NA

I confirm that I have read this submission and believe that I have an appropriate level of expertise to confirm that it is of an acceptable scientific standard.

# Peer review: Reviewer response for version 2

**DOI:** 10.21956/openreseurope.17590.r34105

**Published:** 2023-08-10

**Authors:** Anne-Iris Romens

**Affiliations:** 1 University of Milan - Bicocca, Milan, Italy

I thank the author for having taken my observations into account. I do not have any further comments.

If any, are all the source data and materials underlying the results available?

No source data required

Is the work original in terms of material and argument?

Yes

Is the work clearly and cogently presented?

Partly

Is the argument persuasive and supported by evidence?

Yes

Does the research article contribute to the cultural, historical, social understanding of the field?

Yes

Does it sufficiently engage with relevant methodologies and secondary literature on the topic?

Yes

Reviewer Expertise:

My areas of expertise that relate to the article are migration and gender studies, qualitative methods, as well as decolonial and intersectional approaches.

I confirm that I have read this submission and believe that I have an appropriate level of expertise to confirm that it is of an acceptable scientific standard.

# Peer review: Reviewer response for version 1

**DOI:** 10.21956/openreseurope.16355.r30880

**Published:** 2023-06-01

**Authors:** Lucia Miranda Leibe

**Affiliations:** 1 Universidad Católica Silva Henríquez, Santiago, Chile

The work is undoubtedly a contribution to the theory and literature in the field. It demonstrates a great understanding and mastery of the subject matter, as well as the applied methodology.

 However, the work loses strength and explanatory capacity due to a certain degree of confusion in the structure of presentation.

 Therefore, the following suggestions are proposed:

1. Omit citations or references to works that have not yet been published. It is important for the work to have robust support from the existing theory on the subject matter.
2. I have attached a description of a presentation structure that would facilitate the understanding of the conclusions and the overall content of the work: 
  1. The abstract should contain the research question, position itself within the theoretical framework from which the work is approached, describe the data and sources used to answer the question, as well as the cases on which it is based. 
  2. The introduction should directly address how the text contributes to the field and the innovativeness of the methodology used. The aspects of "micro, meso, and macro approaches" should be justified within the introduction before presenting the structure of the paper. 
  3. The literature review and state of the art should only include published works and not references to "upcoming" ones. 
  4. The methodology is recognized as correct, but it is suggested to emphasize how the overlapping of the observation levels is being avoided. It is also important to justify the contribution of the micro, meso, and macro approaches and why these three levels are chosen instead of focusing solely on one of them in depth. 
  5. The discursive interpretation should be supported by the highlighted theoretical aspects mentioned earlier. 
  6. The conclusions sometimes get lost in the details, and it might be more enriching to simply answer to what extent the personal experiences described by the migrant writers align with what the author of the paper proposes.

If any, are all the source data and materials underlying the results available?

Yes

Is the work original in terms of material and argument?

Yes

Is the work clearly and cogently presented?

Partly

Is the argument persuasive and supported by evidence?

Partly

Does the research article contribute to the cultural, historical, social understanding of the field?

Yes

Does it sufficiently engage with relevant methodologies and secondary literature on the topic?

Yes

Reviewer Expertise:

My area of expertise is the study of feminist and women's movements; and its level and type of internal organization

I confirm that I have read this submission and believe that I have an appropriate level of expertise to confirm that it is of an acceptable scientific standard, however I have significant reservations, as outlined above.

# Response

**Published:** 2023-07-03

**Authors:** Maria Luisa Di Martino

**Affiliations:** Ca' Foscari University of Venice, Italy

1. I have omitted citations to works that have not yet been published.

 2. I have modified following the suggestions made.

 3. I added the contribution

 4. I have modified following the suggestions made.

 5. I think that I have done it in this point, nevertheless I tried to specify the three levels of analysis for each result.

 6.I have modified following the suggestions made.

# Peer review: Reviewer response for version 1

**DOI:** 10.21956/openreseurope.16355.r30885

**Published:** 2023-05-11

**Authors:** Anne-Iris Romens

**Affiliations:** 1 University of Milan - Bicocca, Milan, Italy

The article offers an original analysis that aims at enhancing the standpoint of migrant women in the literature production. Based on the analysis of four novels and interviews with the authors, the paper aims at studying from an intersectional perspective the connection between transnational writings and hybrid identities and put into light the challenges faced by migrant women in their country of immigration. Although the objectives of the paper are of great interest to the scholarly debate, some minor changes are needed to enhance the potential of the contribution.

**1. Introduction**

 The introduction refers to several aims of the paper. However, the contribution would gain clarity by stating more clearly the main argument of the paper, eventually the research questions and how the paper intends to answer them. I would also leave out the information that does not directly connect to the main argument. For instance, I would shorten the reference to the project REWRITE, as the reader loses focus on the paper’s aim.

**2. Literature**

 To highlight the unique perspective of migrant women and how their experience serves as a "mirror for our society" (p3), the author rightly refers to Bhabha (1994)’s writing on colonialism. However, the author refers to ‘universal literature’ only when speaking about Cervantes. The paper would bring a more significant contribution to the debate if it questions, from a decolonial perspective, what is considered legitimate and ‘universal’ culture and what is not.

**3. Methods**

 I found the method of crossing references between books and interviews with the authors very interesting and promising. The reference to ‘migratory career’ was also interesting but came quite late in the text. I would briefly mention it already in the introduction.

 Concerning the sampling, I was quite surprised that a woman born in Italy, whose parents were migrants, was considered in the sample. If the author wishes to maintain the reference to this writer, she needs to specify all along the text that her analysis focuses not only on migrant women but also on migrant descendants, stressing that this specific group is also being ‘otherised’ in European societies.

 I would avoid referring all along the text to the contested concept of ‘second-generation’ but rather refer to that of ‘children of migration’ or ‘migrant descendants’. In this regard, I would also avoid using the terms ‘home country’ or ‘country of origin’ and rather refer to ‘country of birth’ or ‘country of parents’ birth’. Similarly, I would refer to ‘country of residence’ or ‘country of immigration’ rather than to ‘host country’. As the author rightly underlines (p1) transnational literature and migration reshape the concept of ‘home’ and belonging, therefore the ‘home country’ of a migrant can also be where she or he has migrated. I would review the table page 7 in light of these comments.

**4. Findings**

 Although the method was really promising, it is not fully reflected in the finding section. I would avoid having whole paragraphs that do not refer to the selected books and interviews.

 It was not clear to me whether being ‘colonized’ by her mother was a term used by Sabrina (p11). If it was the case, I would make it explicit, otherwise, I would avoid using the expression.

 The author refers to the East-West divide, but I wonder whether countries like Venezuela could be identified as ‘East’, I would provide more arguments for that. Otherwise, the author could refer to other terminologies such as Global North/Global South.

 It was not clear to me what the author understood with ‘pact of honesty with readers’ (p12, p13, p16) and ‘contextual decency’ (p12, p16, p3).

 Based on my assessment, I believe that this article should be accepted (with these revisions) for publication.

If any, are all the source data and materials underlying the results available?

No source data required

Is the work original in terms of material and argument?

Yes

Is the work clearly and cogently presented?

Partly

Is the argument persuasive and supported by evidence?

Yes

Does the research article contribute to the cultural, historical, social understanding of the field?

Yes

Does it sufficiently engage with relevant methodologies and secondary literature on the topic?

Yes

Reviewer Expertise:

My areas of expertise that relate to the article are migration and gender studies, qualitative methods, as well as decolonial and intersectional approaches.

I confirm that I have read this submission and believe that I have an appropriate level of expertise to confirm that it is of an acceptable scientific standard, however I have significant reservations, as outlined above.

# Response

**Published:** 2023-07-03

**Authors:** Maria Luisa Di Martino

**Affiliations:** Ca' Foscari University of Venice, Italy

1. I have added the research question in the introduction and shortened the references to the project.

 2. I agree, but the article is not approached by the decolonial perspective. It would required a total different focus. Nevertheless I have mentioned the importance of the decolonial perspective in the text.

 3. I agree, I have added it in the introduction.

 4. I Have stressed the migrant descendants issue and deleted the term “second generation”

 5. I adopted the term global north and global south along the text.
